# High-Throughput Sequencing for Deciphering the Virome of Alfalfa (*Medicago sativa* L.)

**DOI:** 10.3389/fmicb.2020.553109

**Published:** 2020-09-11

**Authors:** Nicolas Bejerman, Philippe Roumagnac, Lev G. Nemchinov

**Affiliations:** ^1^IPAVE-CIAP-INTA and UFyMA-INTA-CONICET, Córdoba, Argentina; ^2^CIRAD, BGPI, Montpellier, France; ^3^BGPI, INRAE, CIRAD, Institut Agro, Université Montpellier, Montpellier, France; ^4^Molecular Plant Pathology Laboratory, USDA-ARS-BARC, Beltsville, MD, United States

**Keywords:** alfalfa, *Medicago sativa* L., virome, high throughput sequencing, emerging viruses

## Abstract

Alfalfa (*Medicago sativa* L.), also known as lucerne, is a major forage crop worldwide. In the United States, it has recently become the third most valuable field crop, with an estimated value of over $9.3 billion. Alfalfa is naturally infected by many different pathogens, including viruses, obligate parasites that reproduce only inside living host cells. Traditionally, viral infections of alfalfa have been considered by breeders, growers, producers and researchers to be diseases of limited importance, although they are widespread in all major cultivation areas. However, over the past few years, due to the rapid development of high-throughput sequencing (HTS), viral metagenomics, bioinformatics tools for interpreting massive amounts of HTS data and the increasing accessibility of public data repositories for transcriptomic discoveries, several emerging viruses of alfalfa with the potential to cause serious yield losses have been described. They include alfalfa leaf curl virus (family *Geminiviridae*), alfalfa dwarf virus (family *Rhabdoviridae*), alfalfa enamovirus 1 (family *Luteoviridae*), alfalfa virus S (family *Alphaflexiviridae*) and others. These discoveries have called into question the assumed low economic impact of viral diseases in alfalfa and further suggested their possible contribution to the severity of complex infections involving multiple pathogens. In this review, we will focus on viruses of alfalfa recently described in different laboratories on the basis of the above research methodologies.

## Introduction

### Importance of Alfalfa Worldwide

Alfalfa (*Medicago sativa* L.), also known as lucerne, is a major forage crop worldwide cultivated in more than 80 countries, where it is mainly used as silage for grazing livestock (Samarfard et al., 2020). In the United States, it has recently become the third most valuable field crop planted on 22 million acres, with an estimated value of over $9.3 billion (National Alfalfa and Forage Alliance, 2018; Miller, 2019). Argentina is the second largest producer of alfalfa in the world, with alfalfa cultivation covering approximately 17 million acres (Basigalup and Ustarroz, 2007; Miller, 2019). Alfalfa is the principal forage crop in 15 countries of Southern, Eastern and Western Europe, where it is grown on nearly 2.5 million hectares (Julier et al., 2017). Most of these alfalfa fields (65%) are located in Italy, France, Romania, and Spain (Julier et al., 2017).

### Underestimation of Viral Diseases in Alfalfa

Like most agricultural crops, alfalfa is a natural host of many plant viruses (Samac et al., 2015). However, among the groups of pathogens that infect alfalfa, viruses are the least recognized members (Samac et al., 2015; Malvick, 2020). Many, if not all, field management guides for alfalfa growers list acute diseases and pests of the crop without mentioning viral pathogens (Undersander et al., 2011, 2020; Vincelli and Smith, 2014). The reason for this lack of mention is that viruses alone are not capable of killing alfalfa plants and do not appear to cause any major damage or yield losses in the crop. Therefore, they are considered of minor importance to alfalfa production (Samac et al., 2015).

### Multipathogen Infections as a Norm

The field pathology, disease management and studies of host–pathogen interactions in alfalfa, similar to many other plant species and crops, are often limited to a conventional two-way approach involving the host and a single disease-causing biological agent. In natural and cultivated populations, however, plants are frequently infected with a diverse array of pathogens, including many coinfecting viruses, that form multispecies within-host communities (Lamichhane and Venturi, 2015; Tollenaere et al., 2016; Bernardo et al., 2018). The coinfection of plants with different pathogens that may exhibit distinct life cycles, biology and modes of action can alter host susceptibility, affect the severity and dynamics of the disease and create selective pressure, driving the evolution of pathogen virulence (Alizon et al., 2013; Tollenaere et al., 2016; Abdullah et al., 2017). Both symbiotic and antagonistic relationships can occur between microbial species coinfecting a single plant (Lamichhane and Venturi, 2015; Moore and Jaykus, 2018). This is especially true for plant viruses, for which the outcomes of mixed viral infections are unforeseeable and range from coexistence to antagonism (Pruss et al., 1997; Syller, 2012; Elena et al., 2014; Syller and Grupa, 2016; Moreno and López-Moya, 2020). Virus-infected plants, for instance, can be predisposed to secondary infections with non-viral pathogens: the systemic infection of *Arabidopsis thaliana* with cauliflower mosaic virus leads to increased susceptibility to the bacterial pathogen *Pseudomonas syringae* (Zvereva et al., 2016). In contrast, virus-bacteria interactions may have beneficial effects on the host: healthy wild gourd plants (*Cucurbita pepo* ssp. *texana*) were found to contract a bacterial wilt infection at higher rates than plants already infected with zucchini yellow mosaic virus (Shapiro et al., 2013). Overall, the impact of polymicrobial infections on plant health cannot be underestimated. This applies equally to alfalfa, in which the interactions between several pathogens in “disease complexes” are poorly understood (Samac et al., 2015). In most, if not all cases, any association of viral infections with these multispecies consortia is generally unrecognized and, consequently, not assessed in detail. Nevertheless, viral infections of alfalfa represent a ubiquitous and abundant background for all other host–pathogen interactions. Quite reasonably, the same could be true for all *Plantae.*

### HTS as a Tool for the Discovery of New Viruses Infecting Alfalfa

Many of the viruses infecting alfalfa have long been known (Babadoost, 1990; Samac et al., 2015). In recent years, however, a number of new viral pathogens or pathogens that were not previously described in the crop have been discovered (Bejerman et al., 2011, 2015, 2016, 2019; Roumagnac et al., 2015; Nemchinov et al., 2017a, b, 2018a; Kim et al., 2018; Gaafar et al., 2019; Jiang et al., 2019a, b; Samarfard et al., 2020). This became possible due to revolutionary advances in nucleic acid sequencing that have been leading to the replacement of traditional detection methods in plant virology with comprehensive, large-scale, unbiased, reliable high-throughput sequencing (HTS) technologies (Wren et al., 2006; Roossinck, 2012b, 2017; Nagano et al., 2015; Roossinck et al., 2015; Jones et al., 2017; Villamor et al., 2019). The goal of this review is not to address the applications of HTS for the diagnosis and characterization of plant viruses in general because there are numerous good-quality surveys and opinions covering all aspects of this subject in much detail (Barba et al., 2014; Jones et al., 2017; Roossinck, 2017; Maree et al., 2018; Olmos et al., 2018; Villamor et al., 2019). Instead, we will focus on the most recent developments that contributed to elucidating the alfalfa virome and have been implemented in the crop-specific field of alfalfa virology using HTS and the exploration of public transcriptome data repositories.

## HTS Methodologies

Since no virus genes are universally conserved, virologists have developed metagenomics-based approaches that potentially detect plant viromes without *a priori* information (Roossinck, 2012a; Roossinck et al., 2015). These metagenomics-based approaches have targeted several classes of nucleic acids, including total RNA or DNA, virion-associated nucleic acids (VANA) purified from virus-like particles, double-stranded RNAs (dsRNA), and virus-derived small interfering RNAs (siRNAs) (reviewed in Roossinck et al., 2015). Interestingly, the massive acquisition of transcriptomic and genomic data has paved the way for the recovery of virus sequences hidden within these databases. We will hereafter focus on three HTS methodologies that have targeted different viral nucleic acids extracted from alfalfa plants.

### Small Interfering (si) RNA Method

**FIGURE 1:**
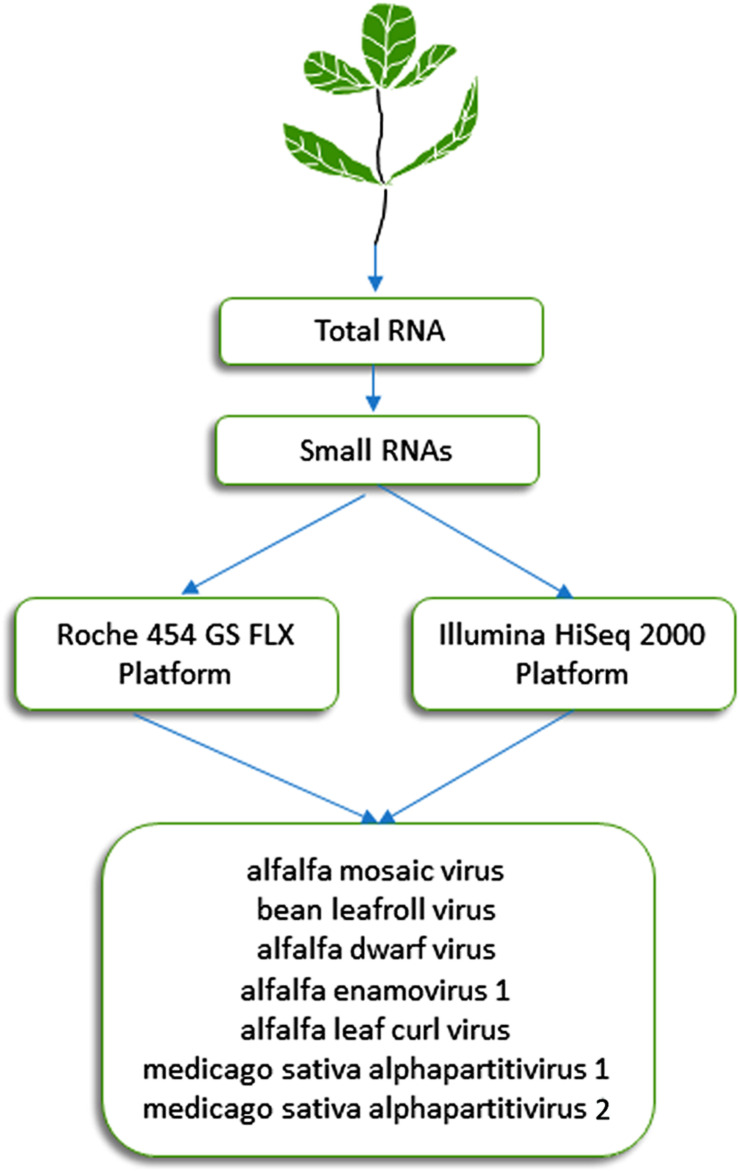
Schematic diagram of the HTS approaches used in Argentina for the identification of seven viruses in alfalfa (Bejerman et al., 2011, 2015, 2016, 2019; Trucco et al., 2014, 2016).

This approach, which was initially described by Donaire et al. (2009) and Kreuze et al. (2009), is based on the analysis of 21- to 24-nucleotide siRNAs that are processed by Dicer-like proteins during the RNA silencing process, which plays a critical role in plant resistance against viruses. It has proven effective during the last decade for detection of known and previously uncharacterized plant viruses (Pooggin, 2018). Specifically, this approach was successful in identifying viruses from alfalfa plants displaying symptoms of alfalfa dwarf disease (Bejerman et al., 2011, 2015), which is prevalent (over 70%) in several growing regions of Argentina and may lead to yield reductions of up to 30% (Lenardon, personal communication). siRNAs extracted from symptomatic alfalfa plants with shortened internodes, a bushy appearance, leaf puckering and varying-sized vein enations on abaxial leaf surfaces were subjected to HTS on an Illumina HiSeq 2000 system (Illumina, United States). This methodology resulted in the identification of six viruses (Figure 1) (Bejerman et al., 2015, 2016, 2018, 2019), which are described below in more detail. The sequencing of siRNAs was also carried out by Guo et al. (2019), who extracted total RNA from pooled alfalfa samples showing different symptomatology using the EASYspin Plant Micro RNA Rapid Extraction Kit and subjected it to the high-throughput sequencing of small RNAs on the Illumina HiSeq 4000 platform. Short reads were assembled into contigs using Velvet 1.0 software. This work resulted in the identification of three known alfalfa viruses (Guo et al., 2019).

### Enrichment of Viral Particles and Virion-Associated Nucleic Acid Sequencing

**FIGURE 2:**
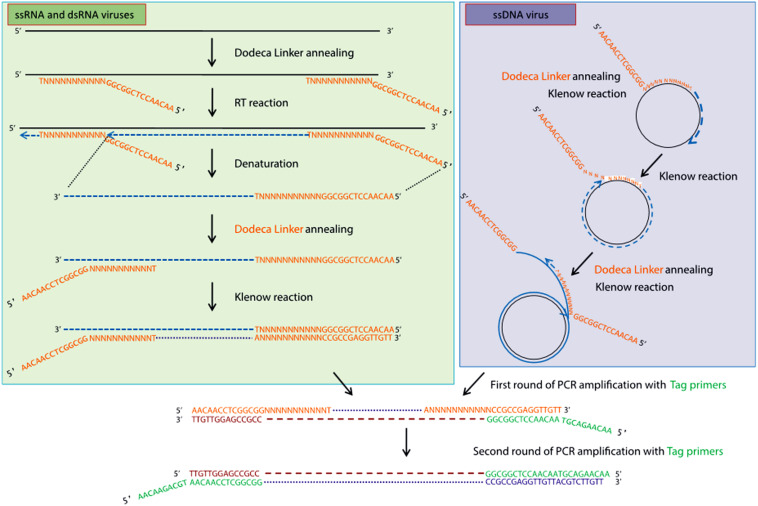
Schematic outline of the VANA-based metagenomics method (François et al., 2018). **Upper left panel:** conversion by the random priming of viral ssRNA or dsRNA with sequence-ready cDNA, including a reverse transcription step, followed by a Klenow reaction step. **Upper right panel:** conversion by the random priming of viral DNA (e.g., circular ssDNA) with sequence-ready cDNA, including a Klenow reaction step (strand displacement amplification). **Bottom panel:** double-stranded DNA is amplified using a single PCR multiplex identifier primer, which yields amplicons that are all tagged at both ends with the same multiplex identifier primer (François et al., 2018). License to publish this figure obtained from the publisher (license #4897140253092).

High-throughput sequencing approaches allowing the enrichment of viral sequences and, thus, improving the sensitivity of virus detection, have also gained popularity during the last decade for inventorying plant virus diversity (Bernardo et al., 2018; Ma et al., 2019). One of these strategies is the VANA metagenomics-based approach (Candresse et al., 2014; Filloux et al., 2015; François et al., 2018), which was successfully used to screen and identify alfalfa viruses in France (Roumagnac et al., 2015). VANA is a combination of several technical steps leading to the concentration of viral particles. These steps include filtration, differential centrifugation and the removal of non-encapsidated material by DNase and RNase digestion. Encapsidated viral DNA and RNA are then extracted from enriched viral particles, RNA is converted to cDNA using a 26 nt primer (Dodeca Linker), and double-stranded (ds) DNA is synthesized using large (Klenow)-fragment of DNA polymerase (Figure 2). dsDNAs are further PCR amplified, and the amplicon libraries are sequenced on either the 454 GS FLX Titanium platform (Bernardo et al., 2018) or the Illumina MiSeq platform as 2 × 300 bp paired-end reads (Nemchinov et al., 2018a). Viral genomes are assembled using Spades (François et al., 2018), CLC Genomics Workbench (Candresse et al., 2014), IDBA-UD (Peng et al., 2012; Ma et al., 2019), CAP3 (Huang and Madan, 1999; Bernardo et al., 2018), and other assemblers. Another approach allowing the enrichment of viral sequences was carried out by Samarfard et al. (2020), who applied the dsRNA immunocapture technique (Blouin et al., 2016) to characterize the virome of alfalfa plants in Australia. dsRNA was captured with specific monoclonal antibodies prior to HTS on an Illumina platform. This work led to the identification of several known alfalfa viruses and the discovery of a new emaravirus tentatively named alfalfa ringspot-associated virus (Samarfard et al., 2020).

### Standard Protocols With Total Plant RNA

Conventional procedures for HTS utilizing total plant RNA have been shown to be efficient for the identification of alfalfa viruses (Nemchinov et al., 2015, 2017a,b; Gaafar et al., 2019). Total RNA is generally extracted from alfalfa samples using the TRIzol RNA isolation reagent (Thermo Fisher Scientific, United States), innuPREP RNA Mini Kit (Analitik Jena AG, Germany) or the RNAeasy Plant Mini Kit (Qiagen, MD, United States) as described by the manufacturers. RNA-Seq libraries are prepared from total RNA using a selection of polyadenylated RNA transcripts for the removal of ribosomal RNA and to obtain higher coverage and better accuracy (Zhao et al., 2018; Gaafar et al., 2019). High-throughput sequencing assays are outsourced and performed on an Illumina platform. Similarly to other protocols, complete genomes are obtained by the assembly of paired-end reads using tools such as SPAdes (Bankevich et al., 2012), Velvet (Zerbino and Birney, 2008), Geneious software (Biomatters Limited, Auckland, New Zealand) or a Qiagen CLC Genomics Workbench and by mapping to the reference genomes, when available, using the Bowtie 2 aligner (Langmead and Salzberg, 2012). Assembly first and mapping-first approaches are usually alternated, depending on the number of short reads and the availability of annotated reference genomes.

## Using Public HTS Repositories as an Open Source for the Discovery of Viral Pathogens

HTS in general, including not only technologies specifically focused on virome research but also those fine-tuned for profiling transcriptional activity in biological systems, provides an immense amount of raw sequencing data. In many cases, the scientific interests of the original submitters of sequences are limited to their respective fields of study, which are frequently unrelated to the discovery of novel transcripts. In other words, only a small portion of the deposited data is subjected to scientific scrutiny, often leaving a considerably larger amount of information untouched, unknown and freely available to the research community. For virologists, this leads to exciting opportunities for the exploration and retrieval of new viral genomes, thus improving the understanding of the diversity and host range of these pathogens and providing essential tools for their diagnosis and characterization. Unsurprisingly, public repositories have become an invaluable tool for the discovery of new pathogens, particularly viral sequences (Bekal et al., 2014; Mushegian et al., 2016; Nibert et al., 2016; Kim et al., 2018; Debat and Bejerman, 2019; Gilbert et al., 2019; Jiang et al., 2019a, b; Lauber et al., 2019; Vieira and Nemchinov, 2019). Computational pipelines for the identification of viral sequences in transcriptomic datasets are numerous and range from custom software packages (Ho and Tzanetakis, 2014; Li et al., 2016; Yamashita et al., 2016; Rott et al., 2017; Zhao et al., 2017; Zheng et al., 2017) (which are often not available for download, may not have an online interface or be regularly updated, or may require the creation of an account with the software providers) to various combinations of freely available short read assemblers, sequence-mapping algorithms and queries for virus-specific domains (Kreuze et al., 2009; Ho and Tzanetakis, 2014; Lambert et al., 2018; Massart et al., 2019). For the purpose of this review, we will discuss in more detail an uncomplicated pipeline used in one of our laboratories for the retrieval of viral sequences from public alfalfa datasets (Jiang et al., 2019a).

**FIGURE 3:**
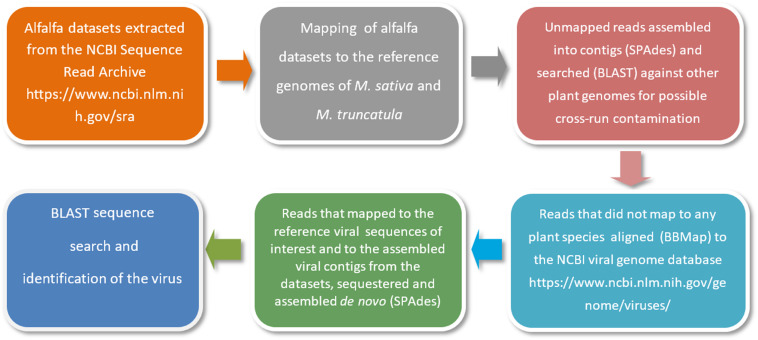
A simplified illustration of the bioinformatics pipeline used for the identification of viral pathogens in alfalfa transcriptome datasets (Jiang et al., 2019a).

As a first step, alfalfa transcriptomic datasets are retrieved from the NCBI Sequence Read Archive (SRA)^1^. The raw sequencing reads are then mapped to the reference genomes of *Medicago sativa*^2^ and a close relative of alfalfa with an annotated genome, *Medicago truncatula*^3^. This is necessary to locate and remove host-derived sequences (host filtering step). Read mapping is usually performed with Bowtie 2 tool (Langmead and Salzberg, 2012). Those reads that are not mapped to the reference genomes are further assembled into contigs using the SPAdes open source software (Bankevich et al., 2012) and searched by BLASTn with default settings and customized parameters (Jiang et al., 2019a) against other plant genomes for a second time to ensure that there is no possible cross-run contamination. Reads that are not mapped to any plant species are next aligned to the NCBI viral genome database, a public resource for virus genome sequence data^4^. Alignments are performed using BBMap^5^, DNASTAR SeqMan NGen software^6^ and Bowtie 2 with very sensitive settings (Jiang et al., 2019a). The very sensitive settings improve the search effort of Bowtie 2 by increasing the cutoffs for which Bowtie will stop searching. The reads that are mapped to the reference viral sequences related to the viruses of interest and to the assembled viral contigs from the datasets are then sequestered and assembled *de novo* using SPAdes (Bankevich et al., 2012). A simplified illustration of the bioinformatics pipeline is presented in Figure 3.

## Alfalfa Virome: Recent Developments

### Previously Known but Not Fully Characterized Alfalfa Viruses Identified by HTS

#### Alfalfa Mosaic Virus

**FIGURE 4:**
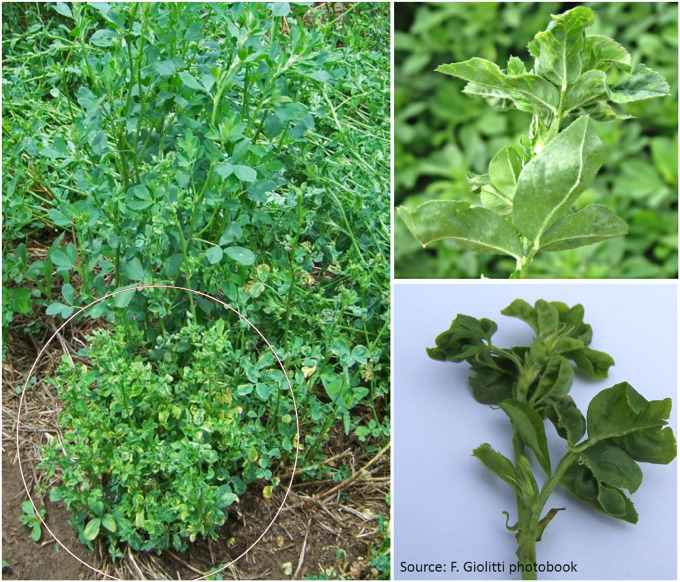
Alfalfa plants exhibiting the alfalfa dwarf disease complex (shortened internodes, bushy appearance, chlorosis at the margins and ribs of the leaflets, leaf puckering and vein enations of varying sizes on abaxial leaf surfaces) (Bejerman et al., 2011).

Alfalfa mosaic virus (AMV) is one of the most important plant viruses distributed worldwide, with a very broad host range (Maina et al., 2019). Despite the large amount of data accumulated on AMV, the application of HTS was necessary to obtain the first complete nucleotide sequence of AMV isolated from alfalfa as a natural host (Trucco et al., 2014). Diseased alfalfa plants exhibited shortening of the internodes, chlorosis at the margins and ribs of the leaflets and vein enations of varying sizes on their abaxial surfaces (Figure 4). Total RNA was purified from enriched viral particles (Figure 1) and used as a template to build libraries that were sequenced on the Roche 454 GS FLX platform (Trucco et al., 2014). The isolate of the virus, designated AMV-Argentina (AMV-Arg), shared a high identity with and presented a similar genome structure to AMV isolates infecting other hosts (Trucco et al., 2014). It is worth noting that the symptoms described above, referred to as alfalfa dwarf disease, were caused by coinfection with several other viruses, as shown below (Bejerman et al., 2011, 2015, 2016, 2018, 2019; Trucco et al., 2016). More recently, complete genomes of two novel AMV isolates from alfalfa plants were obtained in Australia (Samarfard et al., 2020) and China (Guo et al., 2019) employing enrichment of viral nucleic acids prior to HTS and siRNA sequencing, respectively.

#### Bean Leaf Roll Virus

Bean leaf roll virus (BLRV), a member of the genus *Luteovirus* (family *Luteoviridae*), is a phloem-limited virus that has been reported to infect a wide range of legume species worldwide, including alfalfa (Van Leur and Kumari, 2011). It was not diagnosed in Argentina in alfalfa or other crops prior to a study by Trucco et al. (2016). BLRV was isolated from alfalfa plants displaying symptoms of dwarf disease complex (Figure 4) that were coinfected with other viruses. HTS was employed to obtain the first complete nucleotide sequence of BLRV isolated from alfalfa as a natural host. Deep sequencing was performed on the Illumina HiSeq 2000 platform using siRNAs as a template. The complete genome of the BLRV isolate from Argentina (Manfredi BLRV-Arg) was highly identical to BLRV isolates infecting other legume hosts and presented a similar genome structure (Trucco et al., 2016). BLRV-Arg showed a prevalence of over 50% and a wide distribution in Argentinian alfalfa fields.

### Alfalfa Latent Virus

**FIGURE 5:**
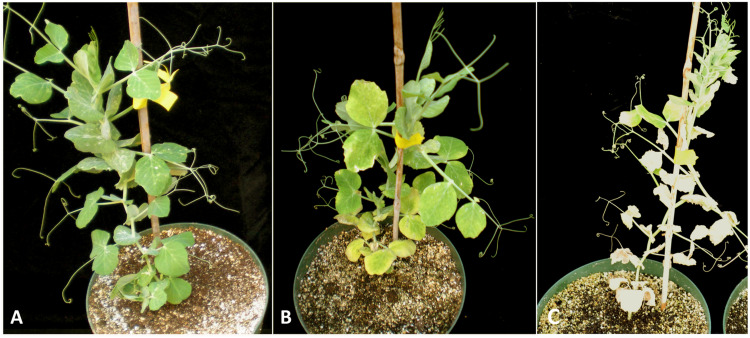
Symptoms of alfalfa latent virus on pea plants (*Pisum sativum*) (Nemchinov, 2017). **(A)** Control uninfected plant. **(B)** Plants infected with ALV showing symptoms of a chlorotic mosaic pattern and necrotic zones along the leaf margins. **(C)** Plants infected with ALV developed extensive necrosis and severe browning (Nemchinov, 2017). License to publish this figure obtained from the publisher (license #4897140253092).

Alfalfa latent virus (ALV) was first reported as a distinct species and a new member of the carlavirus group (Veerisetty and Brakke, 1977). It was subsequently recognized as a strain of *Pea streak mosaic virus*, genus *Carlavirus*, family *Betaflexiviridae* (Hampton, 1981). In the United States, pea streak mosaic virus (PeSV) is common in Nebraska and Wisconsin (Veerisetty, 1979). Although PeSV was first described in 1938 (Zaumeyer, 1938) and ALV was first described in 1977 (Veerisetty and Brakke, 1977), no complete genomic sequences of PeSV and its alfalfa latent strain were available prior to 2015. The first complete genomic sequence of the virus, determined using HTS and primer walking techniques, showed substantial differences from other members of the genus *Carlavirus* (Nemchinov et al., 2015). In addition to a low nucleotide identity to the most closely related species from the genus, *Shallot latent virus* (48.5%, PASC tool, NCBI^7^), the ALV genome did not appear to encode a typical-for-carlaviruses 3′ proximal, cysteine-rich protein of ∼11–16 kDa (Martelli et al., 2007). Instead, it encoded a hypothetical protein of ∼6 kDa that was different from the putative nucleic-acid binding proteins of known carlaviruses. A similar genome structure was later reported for another isolate of PeSV that was 77.9% identical to the alfalfa strain of the virus at the nucleotide level and originated from an unknown host (Su et al., 2015). To confirm that the genomic sequence of ALV was accurate, an infectious cDNA clone of the virus was constructed (Nemchinov, 2017). Rub inoculation of pea plants (*Pisum sativum*) with transcripts generated from the cDNA clone resulted in symptom development within 3 weeks after inoculation. The inoculated plants exhibited chlorotic leaves with noticeable mosaic patterns and necrotic zones along the leaf margins (Figure 5). The plants subsequently showed a decline, developing extensive necrosis and severe browning. When viral preparations purified from the transcript-inoculated pea plants were used to inoculate alfalfa, the alfalfa plants became infected, and the virus was detected in non-inoculated leaves by western blotting with PeSV antiserum, virus-specific RT-PCR and transmission electron microscopy (Nemchinov, 2017). Although ALV is asymptomatic in alfalfa, it can be transmitted mechanically or by aphids to other crops in which it produces symptoms (Veerisetty and Brakke, 1977). This makes alfalfa a natural reservoir of the virus.

### Novel Alfalfa Viruses Identified and Characterized by HTS

#### Alfalfa Dwarf Virus

In 2011, a fragment of the polymerase gene of a cytorhabdovirus was amplified from alfalfa samples showing alfalfa dwarf disease symptomatology by RT-PCR assays with degenerate primers for conserved regions of plant rhabdovirus polymerase (L) genes (Figure 4) (Bejerman et al., 2011). The pathogen, designated alfalfa dwarf virus (ADV), was the first cytorhabdovirus reported to infect alfalfa (Bejerman et al., 2011). Since most of the genes of plant rhabdoviruses described so far have been highly divergent, the application of HTS was essential to elucidate the genomic sequence of ADV. Using siRNA as a template to build libraries to be sequenced on the Illumina HiSeq 2000 platform, the complete genomic sequence of ADV was obtained (Bejerman et al., 2015). The genome of ADV encoded six proteins characteristic of all cytorhabdoviruses as well as one accessory protein, which was also described in ADV-related viruses (Bejerman et al., 2015). The subcellular localization of each ADV-encoded protein, determined by means of transient expression as fusions with green fluorescent protein in the leaves of *Nicotiana benthamiana*, showed that ADV is an unusual rhabdovirus that combines the properties of both cytoplasmic and nuclear rhabdoviruses (Bejerman et al., 2015). Interestingly, the HTS of small RNAs from alfalfa samples collected in Henan Province, China, displaying symptoms such as dwarfism, shrinkage and mottle mosaic revealed fragments of the ADV genome (Guo et al., 2019). Moreover, an analysis of the transcriptome generated in this work, which is publicly available from the NCBI (SRA057663), resulted in the assembly of a novel ADV genome (Humberto Debat, personal communication), thus confirming that this virus was present in China. Furthermore, a contig related to ADV was identified by Jiang et al. (2019a) in the publicly available SRR7252502 alfalfa transcriptome dataset, which was deposited at the NCBI by researchers from Beijing Forestry University in China. Therefore, the use of HTS was critical to determine the complete genome sequence of ADV; it also raised questions about the evolution and the extant geographic distribution of the virus.

#### Alfalfa Enamovirus

The deep sequencing of small RNAs isolated from alfalfa samples showing dwarfism disease (Figure 4) also resulted in the identification of a novel enamovirus, designated alfalfa enamovirus 1 (AEV-1) (Bejerman et al., 2016). AEV-1 was the first enamovirus reported to infect alfalfa. It has a genomic structure characteristic of other known legume-infecting enamoviruses and is phylogenetically related to them (Bejerman et al., 2016; Debat and Bejerman, 2019).

In 2017, the HTS of total RNAs extracted from alfalfa plants collected in Sudan resulted in the assembly of the complete genome of a novel AEV-1 strain, which was designated alfalfa enation virus 2 (AEV-2) (Nemchinov et al., 2017b). At the nucleotide level, AEV-2 was 95.3% identical to AEV-1 from Argentina; its amino acid identity to AEV-1 varied from 94 to 98% for different viral proteins. Phylogenetic analyses of the predicted RNA-dependent RNA polymerase (RdRp) amino acid sequences and the complete nucleotide sequences of AEV-2 and other members of the family *Luteoviridae* clustered AEV-2 and AEV-1 together (Nemchinov et al., 2017b). Although the exact origin of the Sudanese isolate of alfalfa enamovirus is not known, it is possible that its evolution and dissemination into new areas are related to the geography of the host. The first occurrence of the virus outside of Argentina indicated that it might be widespread and can potentially emerge in Iran and southwestern Asia, the geographic origin of alfalfa (Brough et al., 1977), as well as in other alfalfa cultivation regions worldwide.

#### Alfalfa Leaf Curl Virus

**FIGURE 6:**
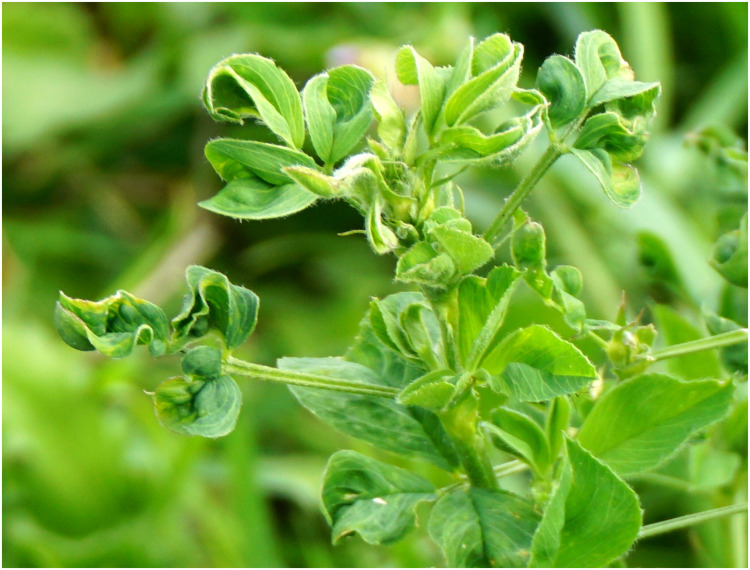
Symptoms of alfalfa leaf curl virus on alfalfa (Roumagnac et al., 2015).

Using a virion-associated nucleic acid (VANA)-based metagenomics approach described above (Candresse et al., 2014), a novel single-stranded DNA (ssDNA) virus designated alfalfa leaf curl virus (ALCV) was identified in alfalfa samples (Roumagnac et al., 2015). ALCV was discovered in plants collected in Southern France that exhibited leaf curl symptoms (Figure 6). The species *Alfalfa leaf curl virus* belongs to the recently designated genus *Capulavirus* within the *Geminiviridae* family. It is unique among geminiviruses in that it is aphid transmitted (Roumagnac et al., 2015). More recently, the siRNA-based HTS of alfalfa samples displaying symptoms of dwarfism disease in Argentina and symptoms of dwarfism, shrinkage and mottle mosaic in China resulted in the identification of novel ALCV isolates (Bejerman et al., 2018; Guo et al., 2019) belonging to ALCV genotype D, which is divergent from three other genotypes (A, B, and C) found in many European countries, Northern Africa and the Middle East (Davoodi et al., 2018). Thus far, one hundred and twenty complete ALCV genome sequences have been recovered from ten countries, and four ALCV genotypes (ALCV-A, ALCV-B, ALCV-C, and ALCV-D) have been clearly distinguished (Davoodi et al., 2018). The identification of these isolates expanded the known geographical range of ALCV and shed more light on the distribution of this emergent alfalfa virus. ALCV isolates were found to be highly recombinogenic and it was suggested that recombination has been a determining factor in the origin of the different viral genotypes. The ALCV sequence data support the hypothesis that the virus likely emerged and diversified in the Middle East before spreading to the western Mediterranean basin and Argentina (Davoodi et al., 2018). The international research conducted on ALCV is a good example highlighting the need to combine the power of HTS for the detection and identification of poorly known viruses with the reliability of classical molecular methodology to obtain complete genomes of novel viruses and understand their evolution (Bernardo et al., 2016).

#### Alfalfa Virus S

**FIGURE 7:**
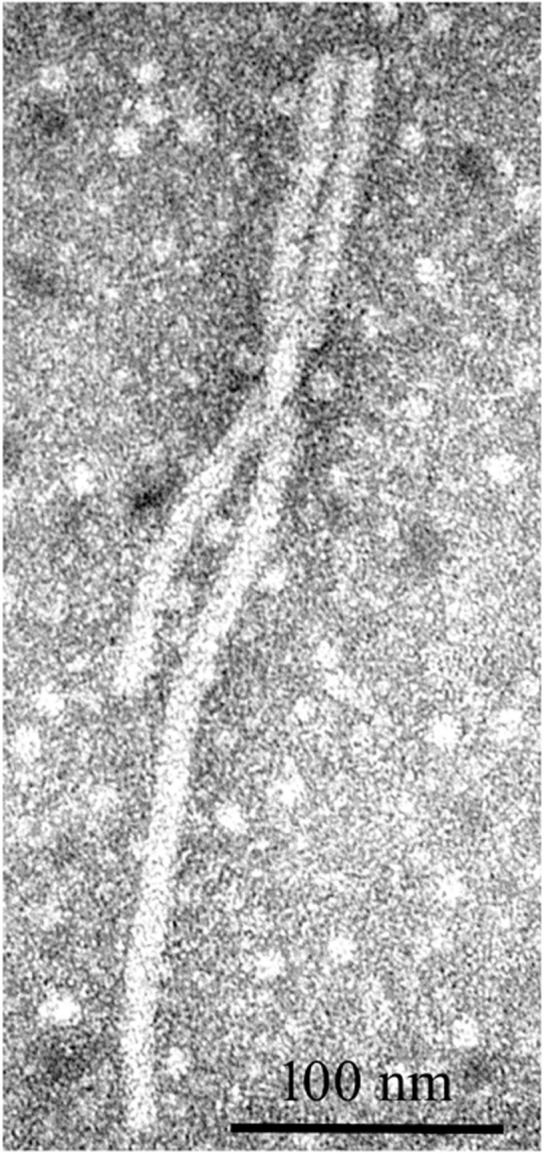
Filamentous virions observed by transmission electron microscopy in alfalfa tissues infected with alfalfa virus S (Nemchinov et al., 2017a).

Alfalfa virus S was discovered in alfalfa samples received from Sudan, Northern Africa, where commercial pivot-irrigated fields were planted with alfalfa seeds originating from the United States (Nemchinov et al., 2017a). Although the plants exhibited chlorosis and stunting in the field, upon arrival at the laboratory, the samples had deteriorated and showed no visual signs of symptoms. Transmission electron microscopy (TEM) observations of the infected tissues revealed the presence of filamentous virions similar to allexiviruses in their length and appearance (Figure 7). The samples were subjected to a standard HTS protocol to detect all viruses that were potentially present in an unbiased manner. Several coding-complete viral genomes were identified in the sequenced sample, including a novel flexivirus with the highest bit score for shallot virus X (ShVX), a virus with ∼98% identity to peanut stunt virus (PSV, genus *Cucumovirus*, family *Bromoviridae*), and a virus with 90–97% identity to alfalfa enamovirus-1 (AEV-1, tentative member of the *Luteoviridae* family) (Bejerman et al., 2016). While PSV and AEV-1 had been known to infect alfalfa, a novel flexivirus, which we referred to as alfalfa virus S, for Sudan (AVS), represented a previously undescribed species. A complete nucleotide sequence of the viral genome consisting of 8,349 nucleotides was obtained by the *de novo* assembly of the HTS-generated reads, supplemented with 5′RACE and the sequencing of the RT-PCR-amplified 3′ terminus (Nemchinov et al., 2017a). At the nucleotide level, AVS was most similar to *Arachis pintoi virus*, *Blackberry virus E* (BVE), and the type member of the *Allexivirus* genus, ShVX. Phylogenetic analyses grouped AVS together with *Arachis pintoi virus* and BVE in a distinct cluster associated with known allexiviruses (Nemchinov et al., 2017a). Similarly to *Arachis pintoi virus* and BVE, the assembled genome of AVS did not appear to have sequences homologous to the 3′ proximal nucleic acid-binding protein of allexiviruses. Currently, *Alfalfa virus S* is recognized by the ICTV as a species in the genus *Allexivirus*.

Recently, two more isolates of the AVS were identified by HTS in alfalfa plants: an isolate from China (GenBank ID MN864567) and an isolate from the USA (GenBank ID MT094142). These results suggest that AVS is more widespread than originally thought. Apart from the fact that AVS often appears to be associated with other pathogens, the economic importance of this virus is largely unknown.

#### Alfalfa Virus F

This novel virus, provisionally designated alfalfa virus F (AVF), was identified using a VANA metagenomics-based approach in symptomless alfalfa samples collected in Southern France (Nemchinov et al., 2018a). The distribution of AVF is currently unknown, and it is unlikely to be restricted to a single area. In accordance with the current species demarcation criteria, the virus represented a distinct species in genus *Marafiviru*s, family *Tymoviridae*. Until 2018, marafiviruses were not known to infect alfalfa. The virus shared the highest degree of sequence identity (∼78%) with medicago sativa marafivirus 1 (MsMV1), which was computationally deduced from alfalfa transcriptomic datasets (Kim et al., 2018). Phylogenetic analysis of the complete nucleotide sequences of AVF and other viruses of the *Tymoviridae* family clustered AVF together with MsMV1 in a sister group connected to the ICTV-proposed marafiviruses: grapevine rupestris vein feathering virus and grapevine Syrah virus-1, thus supporting the classification of AFV as a new member of the *Marafivirus* genus (Nemchinov et al., 2018a).

#### Alfalfa Ringspot-Associated Virus

A novel putative emaravirus, provisionally named alfalfa ringspot-associated virus (ARaV), was recently discovered in Australia (Samarfard et al., 2020) using a dsRNA immunocapture technique (Blouin et al., 2016). Emaraviruses (family *Fimoviridae*, order *Bunyavirales*) have negative-sense, linear, segmented RNA genomes. Only the partial RNA1, 3 and 4 of ARaV, which shared 56–96% amino acid identity with the RdRP, nucleocapsid, and movement (MP) proteins (RNA1, RNA3, and RNA4, respectively) of several emaraviruses, were assembled in the study (Samarfard et al., 2020).

#### Medicago Sativa Alphapartitiviruses

While examining HTS-derived transcriptomic data from two U.S. alfalfa cultivars, cv. Maverick and cv. ZG 9830, it was found that all plants used in the experiment (*n* = 36) contained short reads related to alphapartitiviruses (Nemchinov et al., 2018b). Members of the genus *Alphapartitivirus* infect either plants or ascomycetous and basidiomycetous fungi (Vainio et al., 2018). In plants, partitiviruses cause persistent infections, remaining with their hosts for many generations and having no visible effects on their hosts (Vainio et al., 2018). Plant partitiviruses are transmitted by ovules and pollen to the seed embryo (Boccardo et al., 1987) and are assumed to be mutualistic (Roossinck, 2015). Complete viral genomes were obtained from both cultivars by the assembly of HTS-generated paired-end reads and 5′/3′ rapid amplification of cDNA ends (RACE). The genomes were characteristic of the genus *Alphapartitivirus* and contained two monocistronic segments: double-stranded RNA1 (dsRNA1), encoding an RdRP, and dsRNA2, encoding a viral coat protein (CP). The study was the first to demonstrate that alfalfa cultivars in the United States could be frequently infected with a seed-transmitted cryptic virus of the *Alphapartitivirus* genus.

Alphapartitiviruses were also diagnosed in alfalfa plants with dwarfism disease symptoms in Argentina (Bejerman et al., 2019). HTS of small RNAs isolated from these alfalfa samples led to the identification of two alphapartitiviruses, which were designated medicago sativa alphapartitivirus 1 (MsAPV1) and medicago sativa alphapartitivirus 2 (MsAPV2) (Bejerman et al., 2019). Characterization of the MsAPV1 discovered in this study resulted in the redefinition of the previously reported MsAPV1 genome (Kim et al., 2018), which was likely reconstructed from mixed genomic segments of two different alphapartitiviruses (Bejerman et al., 2019). The MsAPV2 represented a new member of the genus *Alphapartitivirus*, based on the low identity of its CP and RdRp with those of MsAPV1. Thereafter, MsAPV1 was also identified in alfalfa plants originating from Australia (Samarfard et al., 2020). The biological significance and any negative effects of the partitiviruses on alfalfa are currently unknown and require further investigation.

#### Alfalfa-Associated Nucleorhabdovirus

To determine the genome sequence of a rhabdovirus-like pathogen found by electron microscopy in alfalfa samples from Austria, Gaafar et al. (2019) performed high-throughput sequencing followed by RT-PCR to confirm virus infection. HTS was performed on the Illumina MiSeq platform using ribosomal RNA-depleted total RNA as a template. As a result, the authors were able to identify and characterize a new nucleorhabdovirus that shared 39.8% nucleotide sequence identity with its closest known relative, black currant-associated rhabdovirus 1 (Gaafar et al., 2019). This alfalfa-associated nucleorhabdovirus (AaNV) exhibited a unique genomic organization and encoded a new accessory ORF (U) with unknown function located between the matrix and glycoprotein-encoding genes, in addition to the six main nucleorhabdovirus proteins (N, P, P3, M, G, and L) (Gaafar et al., 2019). According to the species demarcation criteria set by the ICTV, alfalfa-associated nucleorhabdovirus (AaNV) represents a new species (Gaafar et al., 2019). AaNV is the first alfalfa-infecting nucleorhabdovirus whose sequence has been characterized. A putative nucleorhabdovirus known as lucerne enation virus (LEV) was previously characterized biologically (Alliot and Signoret, 1972; Leclant et al., 1973). According to its phylogenetic relationships, the AaNV vector could be an aphid, and LEV is likely transmitted by the aphid *Aphis craccivora* (Alliot and Signoret, 1972; Leclant et al., 1973). Therefore, it would be interesting to apply HTS for the characterization of the molecular properties of LEV to determine whether AaNV and LEV represent the same virus species.

## Alfalfa Viruses Found in Public Repositories

### Medicago Sativa Alphapartitivirus 1, Medicago Sativa Deltapartitivirus 1, and Medicago Sativa Marafivirus 1

Kim et al. (2018) analyzed an alfalfa transcriptome dataset downloaded from the NCBI Sequence Read Archive (SRA057663) and identified the genome sequences of three new RNA viruses designated medicago sativa alphapartitivirus 1 (MsAPV1), medicago sativa deltapartitivirus 1(MsDPV1), and medicago sativa marafivirus 1 (MsMV1, belonging to the genera *Alphapartitivirus* and *Deltapartitivirus* of the family *Partitiviridae* and the genus *Marafivirus* of the family *Tymoviridae*, respectively. In this study, high-quality sequence reads were collected using the Sickle program, followed by their assembly into contigs using SPAdes software (Kim et al., 2018). For the identification of the viral sequences, the authors first searched the assembled contigs with BLASTX against a custom database of non-redundant RdRp motifs and then used matched candidates as a query in the NCBI BLASTX search. The RdRp of MsAPV1, MsDPV1, and MsMV1 shared only 68, 58, and 46% amino acid sequence identity with the closest virus species, respectively. The authors concluded that the protocol devised in the study may facilitate the identification of new persistent plant RNA viruses from various plant transcriptome data (Kim et al., 2018). However, as emphasized by Bejerman et al. (2019), who also found MsAPV1 as well as a new alphapartitivirus MsAPV2 in alfalfa HTS repositories, it is critical to pay special attention to the assembly of multisegmented viral genomes when they are obtained from publicly available transcriptomes. The biological significance and any negative effects of the partitiviruses infecting alfalfa are currently unknown and require further investigation.

### Alfalfa Isolate of Cycas Necrotic Stunt Virus

*Cycas necrotic stunt virus*, a member of the genus *Nepovirus*, family *Secoviridae*, was first identified in the gymnosperm *Cycas revoluta* in Japan (Kusunoki et al., 1986; Han et al., 2002). The severely affected plants deteriorated and subsequently died (Kusunoki et al., 1986). Cycas necrotic stunt virus (CNSV) and similar viruses have also been isolated from gladiolus (*Gladiolus* spp.), peony (*Paeonia lactiflora* Pall.), Easter lily (*Lilium longiflorum*), aucuba (*Aucuba japonica*), daphne (*Daphne odora*) and spring onions (*Allium fistlosum*) (Kusunoki et al., 1986; Han et al., 2002; Ochoa-Corona et al., 2003; Hanada et al., 2006; Wylie et al., 2012; Lim et al., 2019; Shaffer et al., 2019). Until recently, CNSV had not been found in alfalfa. CNSV sequences were identified in three NCBI accessions/datasets (SRR7751381, SRR7751384 and SRR7751386) generated from alfalfa plants by third parties using the Illumina HiSeq platform (Dong et al., 2018; Jiang et al., 2019b). The assembled virus had a bipartite (RNA1 and RNA2 segments) single-stranded positive-sense RNA genome that appeared to be coding-complete. Polyproteins P1 and P2, encoded by RNA1 and RNA2 segments, showed 94.3% and 91.3% amino acid identity to the respective polyprotein of the reference CNSV sequence (Jiang et al., 2019b). Phylogenic analyses grouped the alfalfa strain together with CNSV isolated from other species, indicating their origin from the same ancestral virus (Jiang et al., 2019b). Jiang et al. (2019b) concluded that the virus represented a new strain of CNSV adapted to alfalfa, for which the name CNSV-A was proposed. Further research is underway to verify the *in silico* identification of the virus and assess its symptomatology, geographic distribution and economic importance to the alfalfa industry.

### Medicago Sativa Amalgavirus 1

Amalgaviruses are members of the recently established *Amalgaviridae* family that have monopartite double-stranded RNA genomes and encode two proteins: RdRp and CP (Sabanadzovic et al., 2009; Martin et al., 2011; Krupovic et al., 2015). The medicago sativa amalgavirus 1 (MsAV1) sequence was initially communicated by Wang and Zhang in 2013 under GenBank accession number GAFF01077243.1 and subsequently analyzed by Nibert et al. (2016). The virus was not found in the U.S. prior to the study by Jiang et al. (2019a), and its sequence had not been validated experimentally.

**FIGURE 8:**
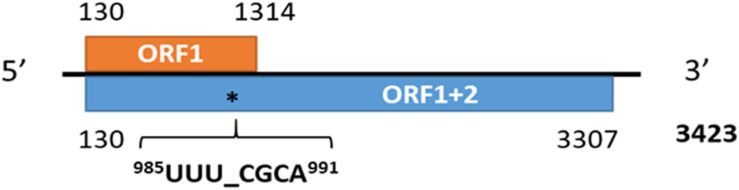
The genome organization of medicago sativa amalgavirus 1 and the putative + 1 programmed ribosomal frameshifting motif in MsAV1 (Jiang et al., 2019a).

The RNA-seq data in which the virus reads were identified originated from the publicly available datasets SRR6050922 to SRR6050957 generated from the U.S. alfalfa cultivars Maverick and ZG9830 (Nemchinov et al., 2017c). The subject of the original study was not related to virology research, and these datasets were evaluated a second time as part of an effort to identify emerging viral genomes in publicly available alfalfa transcriptomic repositories. Among the 36 screened alfalfa datasets, half included MsAV1 reads. The raw viral reads were mapped to the reference genome of MsAV1 (GAFF01077243.1; NC_040591.1) (Zhang et al., 2015; Nibert et al., 2016) and assembled into a complete viral genome (Figure 8). The U.S. isolate of MsAV1 was found to be 100% identical to the GAFF01077243.1/NC040591 isolate from China (Zhang et al., 2015; Nibert et al., 2016) at both the nucleotide and amino acid levels, indicating the same origin of the virus. It is likely that the alfalfa strain of the virus originated in the U.S., since cv. Maverick, used in the study by Zhang et al. (2015), was introduced to China from the United States. In 2020, MsAV1 was also identified in alfalfa plants from Australia (Samarfard et al., 2020). Although the economic significance of amalgaviruses is currently unknown, and with few exceptions, they do not cause any symptoms, amalgaviruses are vertically transmitted through seeds and are persistent in plants. It has been suggested that persistent viruses may represent cytoplasmic epigenetic elements that provide a selective advantage and genetic information to their hosts (Roossinck, 2012a).

### Alfalfa Isolate of Cnidium Vein Yellowing Virus

**FIGURE 9:**
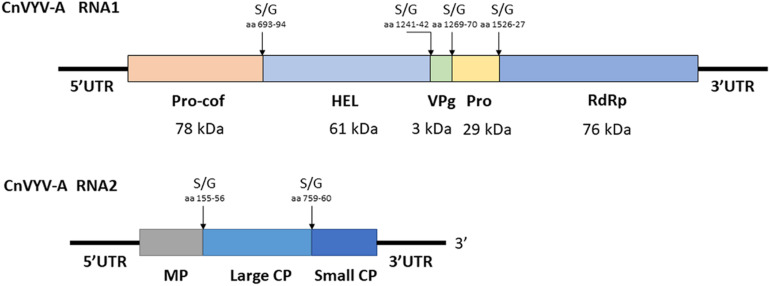
Putative genomic organization of the alfalfa strains of cnidium vein yellowing virus (CnVYV-A). The open reading frames are indicated by boxes, and the putative serine/glycine (S/G) cleavage sites and their amino acid positions are indicated by arrows (Jiang et al., 2019a).

Cnidium vein yellowing virus (CnVYV) is a bipartite, linear, positive-sense ssRNA virus that is a tentative member of family *Secoviridae*, order *Picornavirales* (Yoo et al., 2015a). Two isolates of the virus, CnVYV-1 and CnVYV-2, were previously found to infect cnidium plants (*Cnidium officinale*) in Korea (Yoo et al., 2015a) and no other hosts of CnVYV have been reported. Presently, the virus is not listed by the ICTV as either an established or unassigned species (Thompson et al., 2017). In the study by Jiang et al. (2019a), the datasets were retrieved from the SRA accessions SRR2089795 and SRR2089796 of the BioProject PRJNA289195 (Song et al., 2016) and mapped to the genome of CnVYV (GenBank accession numbers: KR011028, KR011029, KR011030, and KR011031). Both datasets were scanned for possible cross-run contamination, and no plant species other than *Medicago* spp. were detected. The virus reads from both accessions were assembled into coding-complete bipartite genomes consisting of RNA1 and RNA2 segments from two different strains of CnVYV-related viruses. The virus strains were provisionally named CnVYV-A1 and CnVYV-A2 (Jiang et al., 2019a). The RNA1 and RNA2 sequences of the CnVYV-A strains translated into two polyproteins, P1 and P2, which exhibited ∼78–79% (P1) and ∼90–92% (P2) identity to the respective polyproteins of the reference genomes (Jiang et al., 2019a). Based on the predictions made by the Pfam, InterPro and SIAS tools, it was anticipated that CnVYV-A strains share a similar genome organization (Figure 9) with other closely related viruses of the family *Secoviridae* and represent isolates of the same virus strain adapted to alfalfa, for which the name CnVYV-A (alfalfa) was proposed.

### Alfalfa Isolate of Lychnis Mottle Virus

**FIGURE 10:**
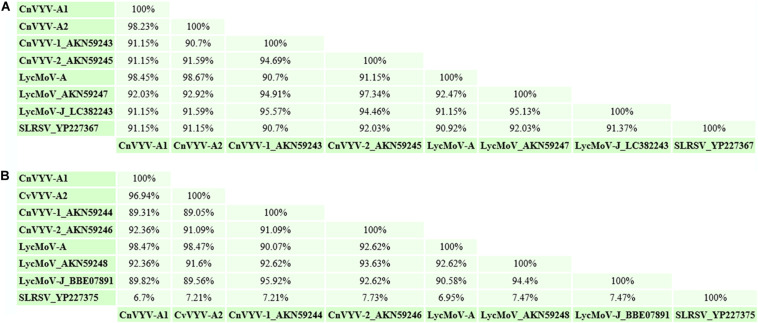
Amino acid identities between the Pro-Pol **(A)** and CP **(B)** regions of the cnidium vein yellowing virus CnVYV-A strains, the CnVYV1 and CnVYV2 strains, lychnis mottle virus LycMoV-A, LycMoV, and LycMoV-J strains and strawberry latent ringspot virus (SLRSV), as predicted by the SIAS tool (http://imed.med.ucm.es/Tools/sias.html) (Jiang et al., 2019a).

Lychnis mottle virus (LycMoV) is a tentative member of the family *Secoviridae* that was first described in *Lychnis cognata*, a flowering plant in the family *Caryophyllaceae* (Yoo et al., 2015b). In 2017, the virus was also isolated from the leaves of *Vincetoxicum acuminatum* in Japan, and the complete nucleotide sequence of LycMoV-J was reported (Fujimoto et al., 2018). While performing a survey of alfalfa transcriptome datasets available at NCBI, transcripts that mapped to the genome of the LycMoV isolate Andong (KR011032 and KR011033) under accession number SRR2089796 (Song et al., 2016) were found (Jiang et al., 2019a). The assembled coding-complete genome of the alfalfa isolate of LycMoV (LycMoV-A) consisted of two segments, corresponding to RNA1 and RNA2 (Jiang et al., 2019a). Putative polyproteins P1 and P2 of LycMoV-A exhibited the top BLAST hits to CnVYV-1 (78.7%) (Yoo et al., 2015b) and LycMoV-J (90.9%) isolates, (Fujimoto et al., 2018), suggesting that CnVYV and LycMoV may potentially belong to the same viral species. To further assess whether LycMoV-A and CnVYV-A are strains of the same virus species or represent different species, as suggested by Yoo et al. (2015a; 2015b) for the Yeongyang isolates of CnVYV from *Cnidium officinale* and the Andong isolate of LycMoV from *Lychnis cognata*, the amino acid identities between their Pro-Pol and CP regions were compared, which are currently used as species demarcation criteria by the ICTV. The SIAS tool^8^ predicted that all the conserved Pro-Pol values were higher than 80% (the ICTV criterion for species demarcation is less than 80% identity), and all the CP values except for that of SLRSV were higher than 75% (the ICTV criterion for species demarcation is less than 75% identity, Figure 10). The authors speculated that CnVYV-A and all the other viruses in the study group (CnVYV-1, CnVYV-2, LycMoV-A, LycMoV and LycMoV-J) with the exception of SLRSV due to the low identity of its CP region, represent individual strains of the same species isolated from different hosts, for which a common name reflecting the taxonomic position and biological characteristics of the species is needed (Jiang et al., 2019a). The following provisional name was suggested for the species: cnidium vein yellowing-like virus (CnVYLV). Phylogenetic analyses of the Pro-Pol region of these viruses between the protease CG motif and the RdRp GDD motif (CG/GDD) in RNA1 fully supported this conclusion (Jiang et al., 2019a).

### Cactus Virus X

**FIGURE 11:**
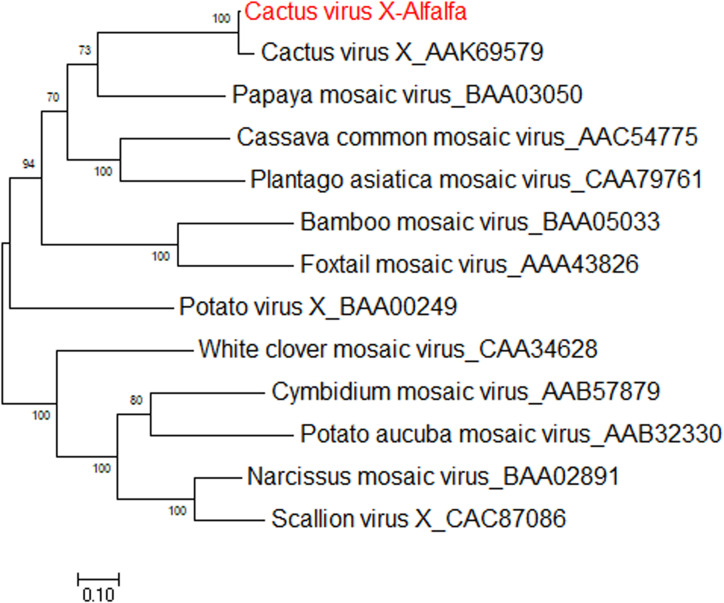
Phylogenetic analyses based on the amino acid alignments of the predicted RdRp sequence of CVX-A and other members of the *Potexviridae* family. The trees were generated using the Maximum Likelihood method of MEGA7 with 1000 bootstrap replicates (Jiang et al., 2019a).

*Cactus virus X* is a member of genus *Potexvirus* (family *Alphaflexiviridae*) that infects various species in the *Cactaceae* plant family worldwide (Koenig, 1987). The reference sequence for cactus virus X (CVX) was reported under two identical accessions, NC_002815.2 and AF308158 (Liou et al., 2004). Prior to the work of Jiang et al. (2019a), CVX had not been identified in alfalfa. CVX-related reads were detected in alfalfa transcriptome dataset SRR7751381, BioProject PRJNA487676 (Dong et al., 2018). The raw reads were assembled *de novo* into a coding-complete genome consisting of a single molecule of linear ssRNA of 6,603 nucleotides in length, excluding the poly(A) tail. At the nucleotide level, the alfalfa isolate of CVX (CVX-A) was 97% identical to the reference sequence (AF308158.2). The CVX-A RNA is translated into five putative open reading frames (ORF) encoding RdRp, triple gene block proteins 1, 2, and 3 and a putative coat protein (Jiang et al., 2019a). It was therefore anticipated that the genome organization of CVX-A is similar to that of CVX (Liou et al., 2004). BLASTP analysis of the amino acid identities of the putative CVX-A proteins with the corresponding proteins of the reference genome indicated a close relationship. Phylogenetic analysis performed with the amino acid sequences of CVX-A RdRp and CP placed CVX-A in the same subcluster with CVX (Figure 11) (Jiang et al., 2019a). Accordingly, it was concluded that CVX-A represents a strain of CVX adapted to alfalfa.

Overall, a systematic survey of more than 600 publicly available alfalfa transcriptome datasets conducted by Jiang et al. (2019a) indicated that approximately 90% of the plant samples employed in the generation of the deposited datasets contained viruses. This analysis identified 23 different viruses, including several emerging viral pathogens that had not been previously reported or experimentally confirmed in *M. sativa*, such as two strains of cnidium vein yellowing virus, lychnis mottle virus and *Cactus virus X*, for which coding-complete genomic sequences were obtained by *de novo* and reference-based assembly (Jiang et al., 2019a). Although further research is needed to confirm the *in silico* identification of these viruses and to specify their symptomatology, geographic distribution and economic importance to the alfalfa industry, the transcriptomic survey improved the knowledge of the host range and diversity and of the viruses infecting alfalfa and provided essential tools for their diagnosis and characterization.

## Concluding Remarks

It is thus becoming increasingly obvious that in research on alfalfa virology, similar to research on the virology of any other plant species or agricultural crop, HTS technologies and their derivatives, such as the exploration of public transcriptomic datasets, are making a major contribution toward the discovery of novel viral genomes, the sequences of emerging pathogens transitioning to new host species and the detection of known viruses. The employment of HTS in the field of alfalfa virology has not only empowered and significantly deepened the understanding of the virome of this strategic legume crop but has also increased the understanding of the geographical range of emergent viruses such as alfalfa dwarf virus, alfalfa enamovirus, alfalfa leaf curl virus, and alfalfa virus S (Table 1). Meanwhile, the virome of alfalfa plants from many other geographic locations, other than those described in this review (Western Europe, United States, China, Australia, and Argentina) remains to be characterized.

**TABLE 1:** Viruses identified in alfalfa by HTS and by analysis of publicly available transcriptome datasets.

Virus name	Genus	Genome	References
Alfalfa mosaic virus	Alfamovirus	ssRNA+	Trucco et al., 2014; Guo et al., 2019; Jiang et al., 2019b; Samarfard et al., 2020
Bean leafroll virus	Luteovirus	ssRNA+	Trucco et al., 2016; Jiang et al., 2019b; Samarfard et al., 2020
Alfalfa dwarf virus	Cytorhabdovirus	ssRNA−	Bejerman et al., 2015; Guo et al., 2019; Jiang et al., 2019b
Alfalfa enamovirus	Enamovirus	ssRNA+	Bejerman et al., 2016; Nemchinov et al., 2017b
Alfalfa leaf curl virus	Capulavirus	ssDNA+	Roumagnac et al., 2015; Bejerman et al., 2018; Guo et al., 2019; Jiang et al., 2019a
Medicago sativa alphapartitivirus 1	Alphapartitivirus	dsRNA+	Kim et al., 2018; Nemchinov et al., 2018b; Bejerman et al., 2019; Samarfard et al., 2020
Medicago sativa alphapartitivirus 2	Alphapartitivirus	dsRNA+	Bejerman et al., 2019; Jiang et al., 2019b
Alfalfa-associated nucleorhabdovirus	Nucleorhabdovirus	ssRNA−	Gaafar et al., 2019
Alfalfa virus S	Allexivirus	ssRNA+	Nemchinov et al., 2017a
Peanut stunt virus	Cucumovirus	ssRNA+	Nemchinov et al., 2017a
Alfalfa virus F	Marafivirus	ssRNA+	Nemchinov et al., 2018a
Medicago sativa marafivirus 1	Marafivirus	ssRNA+	Kim et al., 2018; Nemchinov et al., 2018a
Medicago sativa deltapartitivirus 1	Deltapartitivirus	dsRNA+	Kim et al., 2018; Jiang et al., 2019a
Medicago sativa amalgavirus 1	Amalgavirus	dsRNA+	Nibert et al., 2016; Jiang et al., 2019a; Samarfard et al., 2020
Alfalfa ringspot associated virus	Emaravirus	ssRNA−	Samarfard et al., 2020
Cnidium vein yellowing virus	Unassigned	ssRNA+	Jiang et al., 2019a
Lychnis mottle virus	Unassigned	ssRNA+	Jiang et al., 2019a
Pea streak virus	Carlavirus	ssRNA+	Nemchinov et al., 2015; Jiang et al., 2019a
Cactus virus X	Potexvirus	ssRNA+	Jiang et al., 2019a
Zhuye pepper nepovirus	Nepovirus	ssRNA+	Jiang et al., 2019a
Cowpea mild mottle virus	Carlavirus	ssRNA+	Jiang et al., 2019a
Strawberry latent ringspot virus	Unassigned	ssRNA+	Jiang et al., 2019a
Cycas necrotic stunt virus	Nepovirus	ssRNA+	Jiang et al., 2019b

Needless to say, further disregarding the role of viruses in alfalfa health could be unwise and impractical for alfalfa improvement and production. This is especially true for polymicrobial infections of alfalfa, in which viruses may constitute a substantial and thus far unrecognized part of a disease complex or be solely responsible for coinfections consisting of multiple viral pathogens, as appears to be the case for alfalfa dwarfism disease. Alfalfa may also serve as a natural reservoir for the dissemination of viruses to other agriculturally important crops, although its exact role in the epidemiology of viruses in other crops is not well documented (Babadoost, 1990; Frate and Davis, 2008; Van Leur and Kumari, 2011). Thus, the characterization of the virome of asymptomatic alfalfa plants as well as other *Medicago* species will be crucial to unravel their role as natural viral reservoirs.

Taken together, the continuous discoveries of new viruses in alfalfa made by HTS and associated technologies have called into question the assumed low economic impact of viral diseases in alfalfa and further suggested their potential contribution to the severity of complex infections involving multiple pathogens.

## Author Contributions

All authors listed have made a substantial, direct and intellectual contribution to the work, and approved it for publication.

## Conflict of Interest

The authors declare that the research was conducted in the absence of any commercial or financial relationships that could be construed as a potential conflict of interest.

## Footnotes

1 https://www.ncbi.nlm.nih.gov/sra

2 http://www.medicagohapmap.org/downloads/cadl

3 http://www.medicagogenome.org/

4 https://www.ncbi.nlm.nih.gov/genome/viruses/

5 https://sourceforge.net/projects/bbmap/

6 http://www.dnastar.com/t-nextgen-seqman-ngen.aspx

7 https://www.ncbi.nlm.nih.gov/sutils/pasc/viridty.cgi

8 http://imed.med.ucm.es/Tools/sias.html
